# Tai chi-muscle power training for children with developmental coordination disorder: a randomized controlled trial

**DOI:** 10.1038/s41598-022-25822-x

**Published:** 2022-12-21

**Authors:** Shirley S. M. Fong, Louisa M. Y. Chung, Catherine Mary Schooling, Eric H. Y. Lau, Janet Y. H. Wong, Young-Hyeon Bae, Joanne W. Y. Chung

**Affiliations:** 1 Department of Health and Physical Education, The Education University of Hong Kong, Tai Po, N.T, Hong Kong S.A.R. China; 2 School of Public Health, The University of Hong Kong, Pokfulam, Hong Kong S.A.R. China; 3 Graduate School of Public Health and Health Policy, City University of New York, New York, USA; 4 School of Nursing, The University of Hong Kong, Pokfulam, Hong Kong S.A.R. China; 5 Korea National Rehabilitation Center, Department of Healthcare and Public Health, Rehabilitation Research Institute, Seoul, 01022 Korea; 6 School of Nursing and Health Studies, Hong Kong Metropolitan University, Homantin, Hong Kong S.A.R. China

**Keywords:** Paediatrics, Therapeutics

## Abstract

This study compared the effectiveness of tai chi (TC) muscle power training (MPT), TC alone, MPT alone, and no training for improving the limits of stability (LOS) and motor and leg muscular performance and decreasing falls in children with developmental coordination disorder (DCD). One hundred and twenty-one children with DCD were randomly assigned to the TC-MPT, TC, MPT, or control group. The three intervention groups received TC-MPT, TC, or MPT three times per week for 3 months. Measurements were taken before and after the intervention period. The primary outcomes were the LOS completion time and dynamic LOS scores. The secondary outcomes included the Movement Assessment Battery for Children-Second Edition total test score and percentile rank, knee muscle peak force and time to peak force, and the number of falls. None of the interventions affected the LOS test scores. Improvements in the peak forces of the knee extensors and flexors were demonstrated in the TC (*p* = 0.006) and MPT groups (*p* = 0.032), respectively. The number of falls also decreased in these two groups (*p* < 0.001). Thus, clinicians may prescribe TC or MPT for children with DCD to increase their knee muscle strength and reduce their risk of falls.

## Introduction

Developmental coordination disorder (DCD) is a common neurodevelopmental disorder that affects approximately 6% of primary school-aged children^1^. Children with DCD demonstrate multiple motor deficits, but poor body balance is the most common symptom, with a 73–87% prevalence rate^2^. Poor balance affects motor development, limits participation in activities, and increases the likelihood of falls and injuries^3,4^. Children with DCD do not outgrow their balance difficulties without proper interventions^5^, and therefore, evidence-based treatment strategies are essential.

Although the sensorimotor aspect of balance control in children with DCD has been widely investigated^6–9^, its mechanical aspect, in particular the limits of stability (LOS), is under-examined^3^. The LOS refer to the spatial area in which a standing person can lean without altering his/her foot placement or falling over. It plays an important role in daily activities, such as reaching for objects safely^3^. Recently, our research team found that children with DCD have impaired LOS, and this is associated with a greater number of fall incidents in daily life^3^.

Studies have shown that impaired LOS in children with DCD has two major underlying causes. First, these children have difficulty controlling the movement of their center of gravity (COG), especially when it is close to their LOS^10,11^. This difficulty is primarily associated with atypical patterns of cortical activation and white matter microstructural abnormalities^12^. Second, their reduced leg muscle strength and speed of muscle force production may limit their LOS because they cannot control pelvic or other body movements using their leg muscles in a timely manner when standing^6,7^ and thus may take a corrective step before losing their balance^13,14^.

Exercise training is a standard treatment for children with DCD worldwide^15^. Indeed, it is an enjoyable way of improving balance control of these children. Physiotherapists and parents often send children with motor dysfunctions to participate in exercise training^8,9,16,17^. Tai chi (TC) is a popular Chinese martial exercise and widely acknowledged to improve balance control in older adults^18,19^. TC is particularly suitable for improving COG control and increasing the LOS and leg muscle strength in adults^20–22^. These effects occur because the repeated practice of body weight/COG shifting toward the LOS may enhance plasticity in the brain^23^. Additionally, TC may improve functional motor performance^24^ and prevent falls in older adults^25^. However, because TC uses slow movements, it is unlikely to remediate the reduced leg muscle force production speed that severely affects the LOS and increases the number of fall incidents in children with DCD^6,9^. Therefore, an additional exercise program that could improve leg muscle force production speed and LOS is needed. Previously, we found that muscle power training (MPT) increases the leg muscle force production speed and improves balance in children with DCD^9^ because it maximizes the motor unit firing rate in the initial muscle activation stages^26^ and promotes efferent neural drive to the postural muscles^27^. Thus, when used in conjunction with TC, MPT may be an ideal therapy to improve muscle force production speed and the LOS of balance control in children with DCD. As a result, it may help to improve motor performance and decrease the number of falls in these children.

This study aimed to compare the effectiveness of TC combined with MPT (TC-MPT), TC alone, MPT alone, and no training (control) for improving the LOS of balance control, motor proficiency, and lower limb muscular performance and decreasing the number of falls in children with DCD. We hypothesized that the LOS of balance control in children with DCD is best improved by treating both their suboptimal volitional control of their COG (using TC) and their leg muscular deficits (using MPT). Thus, TC-MPT may be superior to either TC or MPT alone for improving the LOS of balance control and the associated neuromuscular and functional performance and decreasing the number of falls in these children.

## Results

### Study population

**Figure 1:**
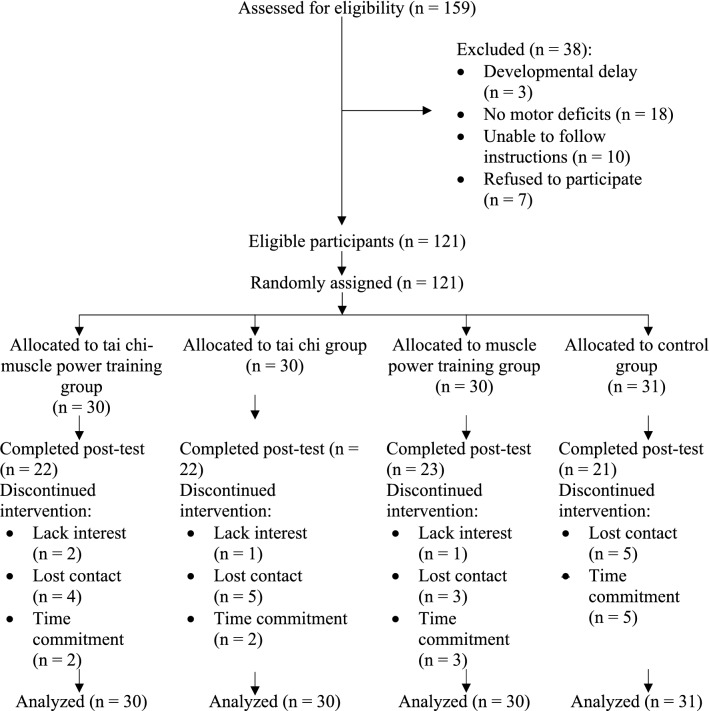
Flow of the study.

From December 2018 to April 2019, 159 children with probable DCD were recruited and screened for eligibility. Of these, 121 qualified for the study and underwent randomization, with 30 assigned to the TC-MPT group, 30 assigned to the TC group, 30 assigned to the MPT group and 31 assigned to the control group. The flow of the participants through screening, randomization, and the interventions is detailed in Fig. 1. The baseline demographics were similar between the four groups with no significant between-group differences (all *p* > 0.05) (Table 1). Note that the ratio for boys to girls was higher than 5 to 1 in our study which was expected as boys have a higher prevalence of DCD when compared to their female counterparts^1^. Nevertheless, the boy: girl ratio was similar between the 4 groups in the present study (*p* = 0.940) (Table 1). In addition, the habitual physical activity level of the MPT group was relatively higher (21.2 ± 51.8 metabolic equivalent hours/week) as compared to the results in other groups. However, there was no significant difference in this demographic data among the four groups overall (*p* = 0.375) (Table 1). Therefore, it was not treated as a covariate in the statistical analysis.

**Table 1:** Baseline characteristics of the participants.

Characteristic	TC-MPT group (n = 30)	TC group (n = 30)	MPT group (n = 30)	Control group (n = 31)	*P*
Age, years	9.5 ± 1.1	9.9 ± 1.2	9.8 ± 1.0	9.7 ± 1.0	0.527
Sex, n	25 boys and 5 girls	26 boys and 4 girls	25 boys and 5 girls	25 boys and 6 girls	0.940
Weight, kg	31.9 ± 9.5	34.9 ± 9.2	32.1 ± 4.7	31.7 ± 6.1	0.304
Height, cm	137.9 ± 11.0	140.5 ± 8.2	140.7 ± 5.1	140.4 ± 10.3	0.584
Body mass index, kg/m^2^	16.5 ± 2.9 (Z score = -0.03 ± 1.11)	17.5 ± 2.6 (Z score = 0.35 ± 0.99)	16.2 ± 1.8 (Z score = -0.13 ± 0.70)	16.1 ± 2.9 (Z score = -0.18 ± 1.11)	0.156
DCD Questionnaire 2007 total score	33.4 ± 11.5	35.4 ± 10.9	33.4 ± 11.0	32.8 ± 10.2	0.808
Habitual physical activity level, metabolic equivalent hours/week	10.6 ± 7.8	12.4 ± 17.0	21.2 ± 51.8	10.3 ± 7.9	0.375
Comorbidity, n (%)					0.499
Attention deficit hyperactivity disorder	3 (10%)	7 (23.3%)	3 (10%)	0 (0%)	
Dyslexia	0 (0%)	6 (20%)	3 (10%)	0 (0%)	
Autism spectrum disorder	7 (23.3%)	11 (36.7%)	7 (23.3%)	0 (0%)	

All values are means ± standard deviations unless specified otherwise.

TC = tai chi, MPT = muscle power training, DCD = developmental coordination disorder.

Twenty-two, 22, 23, and 21 participants from the TC-MPT, TC, MPT, and control groups, respectively, completed the study (Fig. 1). No significant differences were found between the demographic data of the participants who dropped out and those who did not. The average attendance rates at the training sessions were 60%, 61%, and 83% for the TC-MPT, TC, and MPT groups, respectively. All participants complied with the home exercise programs. None of the participants received other exercise or rehabilitation interventions not included in this study.

### Primary outcomes

**Figure 2:**
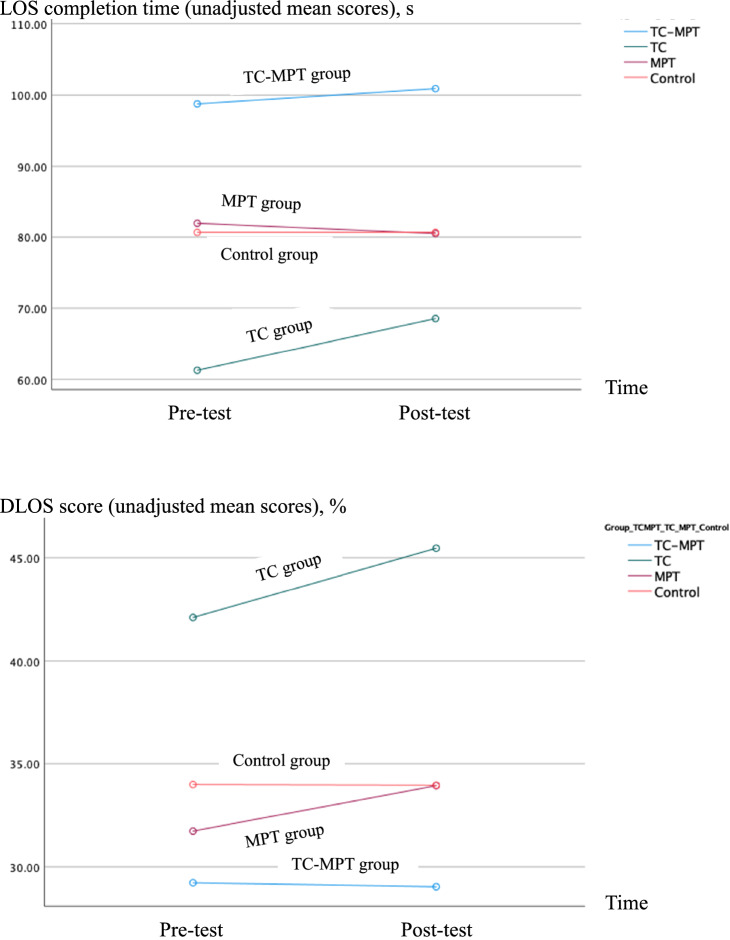
Graphical representation of the primary outcomes. *Note.* Both the LOS completion time and DLOS scores remained relatively stable in all four groups across time. TC = tai chi, MPT = muscle power training, LOS = limits of stability, DLOS = dynamic limits of stability .

Table 2 presents the primary and secondary outcome results in detail. All data was normally distributed as assessed using histograms. There were no group, time, or group-by-time interaction effects in the LOS completion time and dynamic limits of stability (DLOS) scores (all *p* > 0.05). Specifically, the probability values for the group, time and group-by-time interaction effects were 0.867, 0.094 and 0.867, respectively, for the LOS completion time. As for the DLOS scores, the probability values for the group, time and group-by-time interaction effects were 0.250, 0.187 and 0.250, respectively. In addition, both the LOS completion time and DLOS scores remained relatively stable in all four groups across time (Table 2 and Fig. 2), and there were no significant between-group differences at any time points (Table 3).

**Table 2:** Primary and secondary outcomes.

Outcome	TC-MPT group (n = 30)	TC group (n = 30)	MPT group (n = 30)	Control group (n = 31)	Group-by-time interaction effect	Group effect	Time effect
	Mean ± SD	Mean ± SD	Mean ± SD	Mean ± SD	*p*	*p*	*p*
**Primary outcomes**							
**LOS completion time, s**					0.867	0.867	0.094
Pre-test	98.76 ± 34.82 (adjusted mean = 80.66)	61.28 ± 16.13(adjusted mean = 80.66)	81.93 ± 26.78 (adjusted mean = 80.66)	80.66 ± 15.33 (adjusted mean = 80.66)			
Post-test	100.90 ± 39.09	68.54 ± 65.28	80.50 ± 28.05	80.66 ± 2.76			
**DLOS score, %**					0.250	0.250	0.187
Pre-test	29.24 ± 12.72 (adjusted mean = 34.27)	42.11 ± 13.02 (adjusted mean = 34.27)	31.73 ± 8.90 (adjusted mean = 34.27)	33.99 ± 5.06 (adjusted mean = 34.27)			
Post-test	29.04 ± 12.90	45.46 ± 17.52	33.94 ± 14.84	33.96 ± 0.66			
**Secondary outcomes**							
**MABC-2 total test score**					0.993	0.066	0.614
Pre-test	52.17 ± 20.86	63.28 ± 18.39	55.93 ± 16.78	57.13 ± 4.61			
Post-test	51.90 ± 21.22	63.04 ± 18.58	55.70 ± 16.05	57.13 ± 1.90			
**MABC-2 total percentile rank**					0.650	0.650	0.138
Pre-test	14.46 ± 24.14 (adjusted mean = 15.39)	25.05 ± 21.63 (adjusted mean = 15.39)	13.58 ± 18.82 (adjusted mean = 15.39)	8.68 ± 5.19 (adjusted mean = 15.39)			
Post-test	14.45 ± 24.15	25.08 ± 22.16	12.33 ± 17.23	9.00 ± 0.97			
**Peak force of knee extensors, kg**					0.344	0.344	< 0.001*
Pre-test	9.64 ± 3.66 (adjusted mean = 11.07)	12.96 ± 3.37 (adjusted mean = 11.07)	10.61 ± 3.96 (adjusted mean = 11.07)	11.07 ± 1.39 (adjusted mean = 11.07)			
Post-test	9.63 ± 3.47^a^	12.81 ± 3.19^a^	10.93 ± 3.30	11.07 ± 0.58			
**Peak force of knee flexors, kg**					0.028*	0.028*	0.004*
Pre-test	7.44 ± 2.70 (adjusted mean = 7.90)	9.17 ± 2.08 (adjusted mean = 7.90)	7.11 ± 2.30 (adjusted mean = 7.90)	7.90 ± 0.90 (adjusted mean = 7.90)			
Post-test	7.38 ± 2.53^b^	9.81 ± 2.81	8.10 ± 2.97^a^	7.91 ± 1.41^b^			
**Time to peak force of knee extensors, s**					0.800	0.800	0.463
Pre-test	2.30 ± 0.48 (adjusted mean = 2.43)	2.57 ± 0.43 (adjusted mean = 2.43)	2.40 ± 0.28 (adjusted mean = 2.43)	2.43 ± 0.10 (adjusted mean = 2.43)			
Post-test	2.35 ± 0.48	2.77 ± 1.82	2.69 ± 1.73	2.43 ± 0.63			
**Time to peak force of knee flexors, s**					0.651	0.930	0.571
Pre-test	2.34 ± 0.57	2.21 ± 0.60	2.16 ± 0.57	2.23 ± 0.07			
Post-test	2.29 ± 0.53	2.21 ± 0.65	2.39 ± 1.89	2.24 ± 0.37			
**Number of falls in past 3 months**					< 0.001*	< 0.001*	< 0.001*
Pre-test	12.50 ± 32.62 (adjusted mean = 4.66)	1.00 ± 1.82 (adjusted mean = 4.66)	0.47 ± 0.58 (adjusted mean = 4.66)	4.66 ± 5.55 (adjusted mean = 4.66)			
Post-test	5.13 ± 6.16^b,d^	0.77 ± 1.81^a,c^	0.37 ± 0.42^a,c^	4.66 ± 0.82			
**Number of falls in past 12 months**					0.205	0.002*	0.035*
Pre-test	6.44 ± 11.07	3.50 ± 8.94	1.20 ± 1.15	3.71 ± 2.15			
Post-test	2.71 ± 3.18^d^	1.77 ± 2.31^c^	0.87 ± 0.97^c^	3.71 ± 0.68			

All values are means ± standard deviations unless specified otherwise.

Adjusted mean = mean adjusted for baseline differences.

TC = tai chi, MPT = muscle power training, SD = standard deviation, LOS = limits of stability, DLOS = dynamic limits of stability, MABC-2 = Movement Assessment Battery for Children-Second Edition.

*p < 0.05.

Within-group differences:

^a^*p* < 0.05 when compared with pre-test score.

Between-group differences:

^b^*p* < 0.0083 (Bonferroni-adjusted) when compared with the TC group at post-test.

^c^*p* < 0.0083 (Bonferroni-adjusted) when compared with the control group at post-test.

^d^*p* < 0.0083 (Bonferroni-adjusted) when compared with the MPT group at post-test.

**Table 3:** Between-group comparisons of the primary and secondary outcomes.

	TC-MPT vs. TC			TC-MPT vs. MPT			TC-MPT vs. control			TC vs. MPT			TC vs. control			MPT vs. control		
	Mean difference (95% CI)	*p*	*d*	Mean difference (95% CI)	*p*	*d*	Mean difference (95% CI)	*p*	*d*	Mean difference (95% CI)	*p*	*d*	Mean difference (95% CI)	*p*	*d*	Mean difference (95% CI)	*p*	*d*
**Primary outcomes**																		
**LOS completion time, s**																		
Pre-test	37.49 (23.33, 51.64)	< 0.001*	1.38	16.83 (0.78, 32.89)	0.040	0.54	18.11 (4.10, 32.11)	0.013	0.67	-20.66 (-32.08, -9.23)	0.001*	0.93	-19.38 (-27.44, -11.32)	< 0.001*	1.23	1.27 (-9.86, 12.41)	0.820	0.06
Post-test	32.35 (4.55, 60.16)	0.023	0.60	20.40 (2.81, 37.98)	0.024	0.60	20.24 (5.64, 34.84)	0.008*	0.73	-11.96 (-38.19, 14.27)	0.362	0.24	-12.11 (-36.49, 12.26)	0.318	0.26	-0.16 (-10.63, 10.32)	0.976	0.01
**DLOS score, %**																		
Pre-test	-12.88 (-19.53, -6.22)	< 0.001*	1.00	-2.50 (-8.17, 3.17)	0.382	0.23	-4.75 (-9.68, 0.17)	0.058	0.49	10.38 (4.60, 16.16)	0.001*	0.93	8.12 (3.09, 13.15)	0.002*	0.82	-2.26 (-5.95, 1.44)	0.226	0.31
Post-test	-16.42 (-24.37, -8.47)	< 0.001*	1.07	-4.89 (-12.08, 2.29)	0.178	0.35	-4.95 (-9.76, -0.13)	0.044	0.54	11.53 (3.13, 19.92)	0.008*	0.71	11.47 (4.93, 18.02)	0.001*	0.93	-0.05 (-5.60, 5.49)	0.984	< 0.01
**Secondary outcomes**																		
**MABC-2 total test score**																		
Pre-test	-11.11 (-21.27, -0.95)	0.033	0.56	-3.77 (-13.55, 6.02)	0.444	0.20	-4.96 (-12.90, 2.98)	0.212	0.33	7.34 (-1.75, 16.44)	0.112	0.42	6.15 (-0.89, 13.19)	0.085	0.46	-1.19 (-7.65, 5.26)	0.709	0.10
Post-test	-11.14 (-21.45, -0.84)	0.035	0.56	-3.80 (-13.52, 5.92)	0.437	0.20	-5.23 (-13.15, 2.70)	0.188	0.35	7.34 (-1.63, 16.32)	0.107	0.42	5.92 (-1.02, 12.86)	0.092	0.45	-1.43 (-7.42, 4.57)	0.630	0.13
**MABC-2 total percentile rank**																		
Pre-test	-10.58 (-22.43, 1.26)	0.079	0.46	0.89 (-10.30, 12.08)	0.875	0.04	5.79 (-3.40, 14.97)	0.208	0.33	11.47 (0.99, 21.95)	0.032	0.57	16.37 (8.11, 24.63)	< 0.001*	1.04	4.90 (-2.34, 12.14)	0.178	0.35
Post-test	-10.63 (-22.61, 1.35)	0.081	0.46	2.12 (-8.74, 12.99)	0.696	0.10	5.45 (-3.57, 14.47)	0.226	0.32	12.75 (2.49, 23.01)	0.016	0.64	16.08 (7.80, 24.36)	< 0.001*	1.03	3.33 (-3.11, 9.76)	0.299	0.27
**Peak force of knee extensors, kg**																		
Pre-test	-3.31 (-5.13, -1.49)	0.001*	0.94	-0.97 (-2.94, 1.01)	0.331	0.25	-1.43 (-2.87, 0.02)	0.053	0.52	2.35 (0.45, 4.25)	0.016	0.64	1.89 (0.57, 3.20)	0.006*	0.73	-0.46 (-1.97, 1.05)	0.544	0.16
Post-test	-3.17 (-4.89, -1.45)	0.001*	0.95	-1.29 (-3.04, 0.46)	0.145	0.38	-1.44 (-2.73, -0.14)	0.031	0.58	1.88 (0.20, 3.56)	0.029	0.58	1.74 (0.54, 2.93)	0.006*	0.76	-0.14 (-1.38, 1.09)	0.814	0.06
**Peak force of knee flexors, kg**																		
Pre-test	-1.73 (-2.98, -0.49)	0.007*	0.72	0.33 (-0.97, 1.63)	0.614	0.13	-0.47 (-1.52, 0.59)	0.374	0.23	2.06 (0.93, 3.19)	0.001*	0.94	1.26 (0.45, 2.08)	0.003*	0.79	-0.80 (-1.68, 0.09)	0.078	0.45
Post-test	-2.42 (-3.81, -1.04)	0.001*	0.91	-0.72 (-2.14, 0.71)	0.318	0.26	-0.52 (-1.47, 0.42)	0.265	0.26	1.71 (0.21, 3.20)	0.026	0.59	1.90 (0.85, 2.95)	0.001*	0.85	0.19 (-0.92, 1.30)	0.724	0.08
**Time to peak force of knee extensors, s**																		
Pre-test	-0.26 (-0.50, -0.03)	0.028	0.59	-0.10 (-0.30, 0.11)	0.348	0.25	-0.13 (-0.31, 0.05)	0.160	0.37	0.17 (-0.02, 0.35)	0.079	0.47	0.13 (-0.03, 0.30)	0.101	0.45	-0.03 (-0.14, 0.08)	0.563	0.14
Post-test	-0.42 (-1.10, 0.27)	0.231	0.32	-0.34 (-0.99, 0.32)	0.309	0.27	-0.08 (-0.26. 0.10)	0.353	0.14	0.08 (-0.84, 1.00)	0.862	0.05	0.33 (-0.32, 0.99)	0.312	0.25	0.25 (-0.39, 0.90)	0.428	0.20
**Time to peak force of knee flexors, s**																		
Pre-test	0.13 (-0.17, 0.43)	0.396	0.22	0.18 (-0.12, 0.47)	0.239	0.32	0.10 (-0.11, 0.32)	0.344	0.27	0.05 (-0.26, 0.35)	0.764	0.09	-0.03 (-0.25, 0.20)	0.800	0.05	-0.07 (-0.29, 0.14)	0.487	0.17
Post-test	0.09 (-0.22, 0.40)	0.577	0.13	-0.10 (-0.82, 0.62)	0.786	0.07	0.06 (-0.14, 0.25)	0.574	0.11	-0.18 (-0.92, 0.55)	0.616	0.13	-0.03 (-0.28, 0.21)	0.796	0.06	0.15 (-0.55, 0.86)	0.660	0.11
**Number of falls in past 3 months**																		
Pre-test	11.50 (-0.44, 23.44)	0.059	0.50	12.03 (-0.15, 24.22)	0.053	0.52	7.84 (-4.05, 19.74)	0.192	0.34	0.53 (-0.16, 1.23)	0.131	0.39	-3.66 (-5.79, -1.53)	0.001*	0.89	-4.19 (-6.23, -2.14)	< 0.001*	1.06
Post-test	4.37 (1.98, 6.75)	0.001*	0.96	4.77 (2.46, 7.07)	< 0.001*	1.09	0.48 (-1.82, 2.78)	0.675	0.11	0.40 (-0.28, 1.08)	0.244	0.30	-3.89 (-4.57, -3.21)	< 0.001*	2.77	-4.29 (-4.45, -4.13)	< 0.001*	6.59
**Number of falls in past 12 months**																		
Pre-test	2.94 (-2.26, 8.15)	0.262	0.29	5.24 (1.09, 9.40)	0.015	0.67	2.73 (-1.33, 6.79)	0.183	0.34	2.30 (-0.99, 5.59)	0.168	0.36	-0.21 (-3.52, 3.09)	0.897	0.03	-2.51 (-3.40, -1.63)	< 0.001*	1.46
Post-test	0.94 (-0.50, 2.38)	0.194	0.34	1.84 (0.61, 3.08)	0.005*	0.78	-1.00 (-2.19, 0.19)	0.095	0.43	0.90 (-0.02, 1.82)	0.056	0.51	-1.95 (-2.81, -1.09)	< 0.001*	1.14	-2.85 (-3.21, -2.49)	< 0.001*	3.39

TC = tai chi, MPT = muscle power training, CI = confidence interval, d = Cohen’s d, LOS = limits of stability, DLOS = dynamic limits of stability, MABC-2 = Movement Assessment Battery for Children–Second Edition.

*p < 0.0083 (Bonferroni-adjusted).

### Secondary outcomes

An improvement in the peak force of the knee extensors with time was seen exclusively in the TC group, after adjustment for baseline covariates (improved by 1.74 kg, 95% confidence interval [CI]: -2.93 to -0.55, *p* = 0.006). However, the peak force of the knee extensors decreased slightly from baseline to post-test in the TC-MPT group (decreased by 1.44 kg, 95% CI: 0.14 to 2.73, *p* = 0.032; Table 2). In addition, no significant between-group differences in the peak force of the knee extensors were found at post-test (Table 3).

Only the MPT group showed an improvement in the peak force of the knee flexors from baseline to post-test (improved by 0.99 kg, 95% CI: -1.89 to -0.09, *p* = 0.032; Table 2). At baseline, the peak force of the knee flexors was significantly lower in the MPT group compared to the TC group (2.06 kg lower, 95% CI: 0.93 to 3.19, *p* = 0.001), but the mean difference was no longer significant at post-test (mean difference = 1.71 kg, 95% CI: 0.21 to 3.20, *p* = 0.026, Bonferroni adjusted; Table 3).

No significant within-group changes or between-group differences were noted for the time to peak force of the knee extensors and flexors at any time point (all *p* > 0.05, Table 2). The total test score and total percentile rank for the Movement Assessment Battery for Children-Second Edition (MABC-2) were also relatively stable for all groups during the study period. No group, time, or group-by-time interaction effects were noted (all *p* > 0.05, Table 2).

The total number of falls in the past 3 months decreased significantly in both the TC (3.89 falls, 95% CI: 3.21 to 4.57, *p* < 0.001) and MPT groups (4.29 falls, 95% CI: 4.13 to 4.45, *p* < 0.001) from baseline to post-test after adjustment for baseline covariates (Table 2). At post-test, both the TC (mean difference = 4.37 falls, 95% CI: 1.98 to 6.75, *p* = 0.001) and MPT groups (mean difference = 4.77 falls, 95% CI: 2.46 to 7.07, *p* < 0.001) demonstrated less falls than the TC-MPT group (Table 3). A similar pattern of improvement was observed in the total number of falls in the past 12 months (Table 2). At post-test, the TC group had fewer falls than the control group (mean difference = -1.95 falls, 95% CI: -2.81 to -1.09, *p* < 0.001), and the MPT group had fewer falls than the control (mean difference = -2.85 falls, 95% CI: -3.21 to -2.49, *p* < 0.001) and TC-MPT groups (mean difference = 1.84 falls, 95% CI: 0.61 to 3.08, *p* = 0.005; Table 3).

### Adverse events

No adverse events were reported.

## Discussion

This was the first study to compare the effects of TC-MPT with those of TC alone, MPT alone, and no training on the LOS of balance control, motor proficiency, lower limb muscular performance, and fall incidence in children with DCD. There were five major findings. First, only the TC group demonstrated a significant improvement in the peak force of the knee extensors from baseline to post-test, suggesting that as little as 3 months of TC training may strengthen the quadriceps muscles of children with DCD. This finding is not surprising given that TC practice required the participants to maintain a semi-squatting posture while shifting their body weight slowly from one leg to another. This unique movement pattern places a large load on the muscles of the supporting leg and thus stimulates isometric and eccentric contractions of the quadricep muscles. In addition, a wide range of motion of the lower limb joints during TC practice may activate more joint proprioceptors, which may also help develop general muscle strength^28^. In fact, a recent meta-analysis confirmed that TC training effectively increases lower limb muscle strength, and knee muscle strength in particular, in elderly people^22^. TC may also increase leg muscle power in children and adolescents^29^. Our study further verified that TC training may increase the maximum knee extensor muscle strength (peak force) in children with DCD.

However, we found that TC did not improve the time to peak force of the knee extensors in this group of children. Kinetic analysis of TC has revealed that, compared with walking, TC movements have longer peak moment generation times for knee extension^30^. As strength training is velocity-specific^31^, the slowness of the movements used in TC may not be able to shorten the time taken to reach the peak force in the knee extensor muscles of children with DCD. Thus, we suggest that MPT should be incorporated into TC training to improve the knee muscle contraction speed. We found that TC-MPT did not improve the time to peak force of the knee extensors and may even have reduced it after training compared with the baseline value. This finding was unexpected and may have occurred because the post-exercise recovery period was not long enough for metabolic and structural adaptations to occur in the skeletal muscles^32^. Therefore, muscular performance impairment was found in the post-intervention assessment. In future studies, the strength training volume may need to be optimized, and a reasonable recovery period may need to be included before the post-test.

The second major finding was that only the MPT group demonstrated a significant improvement in the peak force of the knee flexors from baseline to post-test. In fact, the results for this metric were similar in the MPT and TC groups after training, indicating that MPT was most effective for improving the peak force of the knee flexors in children with DCD. This finding concurs with the finding of a previous study showing that strength training increases muscle strength in children with DCD^33^ as a result of neuromuscular learning and neural adaptations, such as an enhanced maximal motor unit firing rate^26^ and increased efferent neural drive to the target muscle^27^. Our MPT program included isolated strengthening of the hamstring muscles (Table 4), which was not performed in the other intervention groups. Therefore, the MPT group outperformed the other three groups in improvements in the peak force of the knee flexors. However, the time to peak force did not change after MPT. This may also be attributable to an insufficient recovery period before the post-intervention assessment, as mentioned above.

**Table 4:** The tai chi-muscle power training program to improve center of gravity control and leg muscle force production speed and the limits of stability of balance control in children with DCD.

Type of exercise	Frequency	Intensity	Duration	Aims of exercise
*Warm-up (jogging)*			5 min	
**Tai chi exercises**				
Raising the power	Face-to-face training session: once per week and home practice: twice per week	10 times each	5 min	Challenge standing balance
Withdraw and push			5–10 min	Improve body weight shift in anteroposterior direction, challenge stability limits, and strengthen leg muscles
Wave hands like clouds			5–10 min	Improve weight shift in mediolateral direction and challenge stability limits
Grasp the sparrow’s tail			5–10 min	Improve hip movements and help integrate movements of the legs with the torso and arms
Twist step and brush knee			5–10 min	Maintain dynamic body balance
*Break*			5 min	
**Muscle power training**				
Squatting exercise	Face-to-face training session: once per week and home practice: twice per week	4 sets, 10 repetitions, 70% of 1 repetition maximum (perform bilaterally)	5 min	Strengthen hip and knee extensors and ankle plantar flexors
Hip flexion in standing position			5 min	Strengthen hip flexors
Seated knee extension			5 min	Strengthen knee extensors
Hamstring curls in prone position			5 min	Strengthen knee flexors
Hip abduction/ adduction while lying on side			5 min	Strengthen hip abductors and adductors
Seated ankle dorsiflexion			5 min	Strengthen ankle dorsiflexors
Standing calf raises			5 min	Strengthen ankle plantar flexors
*Cool-down (stretching)*			5 min	

The tai chi-muscle power training group followed the entire protocol, the tai chi group received only the simplified tai chi program, and the muscle power training group received only the muscle power training. All three intervention groups received a total of 90 min of training (repeated practice) per session.

The third major finding was that, in contrast to our hypothesis, MPT and/or TC did not improve the LOS (neither the LOS completion time nor DLOS score) in children with DCD. This finding differs from the finding of a study by Tamplain et al.^34^, which showed that a 10-week group-based gross motor training program improved BioSway™-derived DLOS scores in children with DCD. The major difference between the study of Tamplain et al.^34^ and our study is the intervention activities used. Their intervention included functional balance tasks, such as hopping, skipping, and sit-and-stand activities, with an object or a partner (i.e., task-oriented training), while our intervention included TC and/or specific muscle strengthening exercises aimed at remediating the neuromuscular deficits of children with DCD. Thus, task-specific balance training may be superior to TC and/or MPT at improving the LOS of balance control in children with DCD. Moreover, our TC and MPT interventions did not address the impaired motor inhibition problem^35^ nor strengthen the weak core muscles of these children^36,37^, which may explain the lack of effect on LOS test performance.

In addition to the unchanged LOS performance, the motor proficiency (MABC-2 scores) of children with DCD also showed no change after TC and/or MPT (the fourth major finding). It is known that strength training (i.e., MPT) on its own does not improve dynamic motor performance^38^. However, TC training (6 weeks, 3 days/week, 40 min/session) was shown to improve MABC-derived balance scores in children with autism spectrum disorder (ASD)^39^, and motor skills training (8 weeks, 3 days/week, 60 min/session) improved the performance of both practiced (e.g., balance) and non-practiced motor tasks (e.g., manual dexterity) in children with DCD^40^. Therefore, it was expected that TC and TC-MPT would improve the MABC-2-assessed motor performance. Further studies should explore the reasons why these interventions did not improve motor proficiency in children with DCD.

The final important finding of this study was that the number of fall incidents (in the past 3 and 12 months) decreased significantly after TC or MPT. However, the TC-MPT group did not show any improvement in the number of falls after training. It is widely acknowledged that increasing leg muscle strength and the rate of muscle force development prevents falls in children^41,42^. As leg muscular performance improved in both the TC and MPT groups (Table 2), it is logical that the number of fall incidents also decreased. However, leg muscular performance in the TC-MPT group did not improve after training, as explained above. Thus, the number of fall incidents also remained unchanged in this group. As the etiology of falls is multifactorial and involves extrinsic (environmental) and intrinsic (patient-related) factors^41^, further studies may explore more optimal interventions to improve the LOS of balance control and muscular performance and decrease the number of falls in children with DCD.

This study had several limitations. First, we did not include a follow-up assessment due to the social distancing policy and school closures caused by the COVID-19 pandemic. A follow-up assessment will be set up to evaluate the carryover effects of the interventions in future studies. Second, the participants and their parents were not blinded to the group assignment due to the nature of the exercise training. Thus, the participants in the intervention and control groups may have had different expectations about the treatment outcomes, which may have introduced bias in the results. Third, this was an experimental study of children with DCD aged 9 to 12 years old only. The results may not be generalizable to younger or older children with DCD or those with other neurodevelopmental disabilities. Forth, children in the control group received no training during the study period. Therefore, we cannot confirm whether the beneficial effects demonstrated in the 3 intervention groups were due to exercise training per se or TC and/or MPT. Further studies should incorporate an active control group (receiving general exercise training) to confirm the results. Finally, our study sample included DCD children with comorbidities such as attention deficit hyperactivity disorder, dyslexia and autism spectrum disorder which may have confounded the results. Future studies may include children with DCD only.

## Conclusions

TC training strengthened the knee extensor muscles and MPT strengthened the knee flexor muscles of children with DCD. Either TC or MPT alone effectively decreased the number of falls in this group of children. However, TC and MPT combined did not improve lower limb muscular performance, motor performance, or the LOS and balance control or decrease the number of falls in these children. Thus, clinicians may suggest TC or MPT as stand-alone interventions to children with DCD and their parents.

## Methods

### Design overview

This was a single-blinded, randomized controlled trial with four parallel arms. Clinical trial registration was completed at ClinicalTrials.gov (NCT03598478) on 26/07/2018. Ethical approval was obtained from the Institutional Review Board of the University of Hong Kong (UW 16–507). Written informed consent was obtained from each participant and parent before enrolment in the study. All experimental procedures were conducted in accordance with the Declaration of Helsinki. Figure 1 depicts a Consolidated Standards of Reporting Trials flow diagram of the study.

### Participants

Children with probable DCD were recruited from local primary schools and our research team’s database of DCD patients, using poster and social media advertising and letter invitations. Children were included if they: (1) were 9–12 years old (as cognitive development is more matured, reaching the concrete operational stage according to Jean Piaget, which may be necessary for TC training)^43^; (2) were diagnosed with DCD according to the Diagnostic and Statistical Manual of Mental Disorders V (DSM-5)^1^; (3) had a total MABC-2 test score < 67^44^; (4) had a total score < 55 (for children aged 9 years) or < 57 (for children aged 10 to 12 years) on the DCD questionnaire 2007 (Chinese version)^45^; and (5) attended a mainstream primary school (i.e., intelligence level within the normal range). Children were excluded if they: (1) had any known significant congenital, cognitive, psychiatric (other than comorbid attention deficit hyperactivity disorder or ASD), neurological, sensory, musculoskeletal, or cardiopulmonary disorder that may affect test performance; (2) were receiving active treatment, such as physiotherapy; (3) had prior experience in TC or MPT; (4) demonstrated excessive disruptive behavior; or (5) were unable to follow instructions.

### Screening, randomization, and allocation concealment

The initial screening was performed by two physiotherapists at the University of Hong Kong. Eligible participants were then randomly assigned to the TC-MPT, TC, MPT, or control groups. To ensure concealed allocation, the randomization procedure involved sealed opaque envelopes prepared by an independent researcher, who was not involved in participant recruitment. A random-number table was used to generate the allocation sequence. The corresponding author performed the allocation procedure, including opening the envelopes.

### Interventions

The interventions were conducted in a physical activity room at the University of Hong Kong. Children in the TC-MPT, TC, and MPT groups attended a 90-min training session once per week for 12 weeks. The sessions were supervised by a physiotherapist and conducted by a certified TC and fitness instructor. In addition, home exercises were prescribed to each participant to increase the total training frequency to three times per week. The home exercises (90 min/session, twice/week, 12 weeks) were the same as those practiced in the supervised training sessions. The parents were provided with clear written instructions and a logbook to record the training volume and progress. They were requested to submit the signed logbooks to the assessor after completing the intervention.

### Tai chi-muscle power training group

Participants in the TC-MPT group received two levels of training within each 90-min group-training session: (1) TC training and (2) MPT. The TC training protocol, which was modified from the Harvard Medical School Guide to Tai Chi^46^, is presented in Table 4. It consisted of five basic TC movements designed to improve body balance, weight-shifting ability, stability limits, and leg muscle strength. All participants learned and practiced one to two TC movements in each training session and progressively added movements over the 12-week training period.

After receiving TC training, participants in the TC-MPT took a 5-min break and then received MPT. The MPT protocol was designed and refined by our research team to improve leg muscle strength and the rate of strength development (muscle contraction speed) and minimize postural sway in children with DCD^9^. During MPT, the participants contracted their major postural muscles bilaterally, including the gluteus maximus (squatting), iliopsoas (resisted hip flexion), quadriceps (resisted knee extension and squatting), hamstrings (hamstring curls), gluteus medius and minimus (resisted hip abduction), tibialis anterior (resisted ankle dorsiflexion), and gastrocnemius and soleus (calf raises and squatting) muscles, as fast as possible against a resistance equivalent of 70% of one repetition maximum (RM, Table 4). One RM was defined as the maximum load that could be lifted at one time, and it was estimated in the first session and once every month throughout the intervention period. The training load relative to 1 RM was adjusted, accordingly, as a form of progression^9^.

### Tai chi-only and muscle power training-only groups

Children in the TC group skipped the MPT session and practiced TC movements repeatedly for 90 min in a group format. Those in the MPT group performed group-based strengthening exercises repeatedly for 90 min, with a short 5-min break between the three sets of exercises (Table 4). The exercise progression pattern in the TC- and MPT-alone groups was the same as the progression pattern in the TC-MPT group.

### Control group

Participants in the control group received no intervention within the study period but were allowed to continue their normal daily activities and usual medical care.

### Test procedures and blinding of assessors

All participants were assessed by two blinded assessors before beginning the intervention (baseline or pre-test) and shortly after the intervention period (post-test, Fig. 1). Every participant, regardless of their group assignment, underwent the same assessments in random order in the physical activity laboratory at the University of Hong Kong or the Education University of Hong Kong. To ensure assessor blinding, both the participants and their parents were instructed not to disclose their group assignments to the assessors.

### Demographics

Each participant’s age, sex, comorbid conditions, and medical history were obtained from their medical records, if any, and by interviewing their parents. The body weight and height were measured, and body mass index was calculated by dividing the body weight by the square of the height. The habitual physical activity level was estimated based on the exercise intensity, duration, and frequency and the assigned metabolic equivalent (MET) value of the physical activity with reference to the Compendium of Energy Expenditures for Youth^47^. Motor difficulties were quantified using the DCD questionnaire 2007 (Chinese version)^45^.

### Primary outcomes

#### Limits of stability

A BioSway™ computerized dynamic posturography system (Biodex Medical Systems Inc., Shirley, NY, USA) was used to perform the LOS test. This device has been shown to be valid and reliable when assessing LOS in young individuals (inter-tester intraclass correlation coefficient = 0.70 and intra-tester intraclass correlation coefficient = 0.82)^48^. During the test, each participant kept his/her eyes open and stood barefoot on the force platform of the machine with standardized foot placement. S/he then intentionally leaned his/her body or shifted his/her body weight from the center position to eight spatial targets in random order and then back to the center as quickly, directly, and accurately as possible. The target positions represented the perimeter of the theoretical 100% LOS (advanced skill level), 75% LOS (moderate skill level), and 50% LOS (easy skill level). If a participant was unable to lean through his/her 100% LOS, a lower skill level (e.g., 75% LOS) was used. The displacements of the participants’ center of pressure and the vertical projection of the COG onto the force platform while leaning in different directions were displayed on a screen in real time as visual feedback and recorded automatically. A DLOS score (%) was then derived for each of the eight movement directions, and an overall DLOS score was calculated by averaging the eight DLOS scores. A higher DLOS score (close to 100%) represents a more accurate and direct COG trajectory to the LOS target and back to the center position, reflecting better COG volitional control and LOS-specific balance ability^49^. The total time taken (in seconds) to complete the LOS test, together with the overall DLOS score, were documented for analysis.

### Secondary outcomes

#### Functional motor performance

The MABC**-**2 is a standardized assessment tool commonly used to measure functional motor performance in children aged 3 to 16 years. It has demonstrated good test–retest reliability, inter-rater reliability, and criterion-related validity^44^. The assessment comprises eight functional test items categorized into three domains: manual dexterity, aiming and catching, and balance. The raw score for each test item was converted into an item standard score, component score, component standard score, and component percentile. The total test score (i.e., the sum of the eight item standard scores), total standard score, and total percentile rank were then derived. The total test score and total percentile rank, which reflect the participants’ overall functional motor performance, were used for analysis. A higher total test score or a higher total percentile rank indicate better functional motor performance^44^.

### Leg muscular performance

The muscular performance of two important postural muscles, the quadriceps (knee extensors) and hamstring (knee flexors), was assessed using a Lafayette Manual Muscle Test System (Model 01,165; Lafayette Instrument Company, Lafayette, LA, USA) following standardized manual muscle testing procedures^50^ and dynamometer placements^51^. This muscle assessment method has been shown to have good to perfect reliability (intraclass correlation coefficient = 0.81**–**0.98) in young people with disabilities^52^. Each participant completed a familiarization trial and then a manual muscle test, during which the peak force was generated for 2 s for each muscle group. The participants were instructed to contract their leg muscles as hard and as fast as possible. The peak force of each muscle group in the test was used for analysis. In addition, the time to peak force, defined as the elapsed time from the start of the test until the maximum force was reached^51^, of each muscle group was documented for analysis. A shorter time taken to reach peak force indicates greater muscle force production speed^6^.

### Fall history

A fall is defined as unintentionally coming to rest on the ground, regardless of whether it results in an injury^53^. The participants’ fall histories were obtained through self-reports and parent-reports of the number of falls and the associated injuries, if any, in the past 3 and 12 months^3^. A 12-month recall period was selected as it has been reported that the recall accuracy of the number of falls in the preceding year is satisfactory in adults (sensitivity = 89% and specificity = 95%)^54^. A 3-month recall period was also included as the interventions lasted for only 3 months. The number of falls was included in the statistical analysis.

### Statistical analysis

G*Power software (version 3.1.0; Franz Faul, University of Kiel, Germany) was used to estimate the sample size. In our previous study, children with DCD achieved significant improvements in postural control (primary outcome) after 3 months of functional movement-MPT compared with controls (effect size = 0.73)^9^. Therefore, an effect size of 0.8 was used for the between-group comparison of the primary outcome in this study. To achieve 80% statistical power with the two-tailed alpha level set at 5%, 26 children per group were needed. Anticipating an overall 15% drop-out rate^55^, 30 children with DCD per group were recruited, for a total of 120 children.

SPSS version 27 software (IBM, Armonk, NY, USA) was used to analyze the data based on the intention-to-treat (last observation carried forward) principle. Descriptive statistics were generated for all demographic and outcome variables. Data normality was assessed using histograms, and outliers were removed. Demographic and outcome variables at baseline were compared across groups using a one-way analysis of variance (for continuous data) or a chi-square test (for categorical data). A repeated-measures analysis of covariance (ANCOVA, group × time) was used to compare the outcome variables of the four groups across the two time points, with adjustment for baseline covariates (i.e., LOS completion time, DLOS score, MABC-2 total percentile rank, peak force of the knee extensors, peak force of the knee flexors, time to peak force of the knee extensors, and number of falls in the past 3 months at baseline were treated as covariates in the ANCOVA analysis). Post hoc between-group comparisons were performed using independent Student’s t tests, and within-group changes from baseline to post-test were analyzed by paired t tests, as appropriate. The overall two-tailed significance level was set at 0.05. Bonferroni’s correction was applied for multiple comparisons, as necessary.

## Data Availability

Data from this study are available on request from the corresponding author (smfong_2004@yahoo.com.hk).
